# Enhanced expression of WD repeat-containing protein 35 (WDR35) stimulated by domoic acid in rat hippocampus: involvement of reactive oxygen species generation and p38 mitogen-activated protein kinase activation

**DOI:** 10.1186/1471-2202-14-4

**Published:** 2013-01-07

**Authors:** Koji Tsunekawa, Fumio Kondo, Teruhiko Okada, Guo-Gang Feng, Lei Huang, Naohisa Ishikawa, Shoshiro Okada

**Affiliations:** 1 Department of Pharmacology, Aichi Medical University School of Medicine, 1-1 Yazakokarimata, Nagakute, Aichi, 480-1195, Japan

**Keywords:** Domoic acid, WDR35, Hippocampus, AMPA/KA receptor, ROS, p38 MAPK

## Abstract

**Background:**

Domoic acid (DA) is an excitatory amino acid analogue of kainic acid (KA) that acts via activation of glutamate receptors to elicit a rapid and potent excitotoxic response, resulting in neuronal cell death. Recently, DA was shown to elicit reactive oxygen species (ROS) production and induce apoptosis accompanied by activation of p38 mitogen-activated protein kinase (MAPK) *in vitro*. We have reported that WDR35, a WD-repeat protein, may mediate apoptosis in several animal models. In the present study, we administered DA to rats intraperitoneally, then used liquid chromatography/ion trap tandem mass spectrometry (LC-MS/MS) to identify and quantify DA in the brains of the rats and performed histological examinations of the hippocampus. We further investigated the potential involvement of glutamate receptors, ROS, p38 MAPK, and WDR35 in DA-induced toxicity *in vivo*.

**Results:**

Our results showed that intraperitoneally administered DA was present in the brain and induced neurodegenerative changes including apoptosis in the CA1 region of the hippocampus. DA also increased the expression of WDR35 mRNA and protein in a dose- and time-dependent manner in the hippocampus. In experiments using glutamate receptor antagonists, the AMPA/KA receptor antagonist NBQX significantly attenuated the DA-induced increase in WDR35 protein expression, but the NMDA receptor antagonist MK-801 did not. In addition, the radical scavenger edaravone significantly attenuated the DA-induced increase in WDR35 protein expression. Furthermore, NBQX and edaravone significantly attenuated the DA-induced increase in p38 MAPK phosphorylation.

**Conclusion:**

In summary, our results indicated that DA activated AMPA/KA receptors and induced ROS production and p38 MAPK phosphorylation, resulting in an increase in the expression of WDR35 *in vivo*.

## Background

Domoic acid (DA) is an excitatory amino acid analogue of kainic acid (KA) that acts through glutamate receptors to elicit a rapid and potent excitotoxic response, resulting in prolonged receptor activation, tonic depolarization and neuronal hyperexcitability, excessive Ca^2+^ influx, and ultimately widespread neuronal loss [1,2]. Several studies have reported that the toxicity of DA is mainly mediated by the alpha-amino-3-hydroxy-5-methyl-4-isoxazolepropionic acid (AMPA)/KA receptor in immature and mature neurons and glia from rat cerebellum [3,4]. Furthermore, Giordano et al. [5] reported that an AMPA/KA receptor antagonist, but not an N-methyl-_D_-aspartate (NMDA) receptor antagonist, prevents DA-induced apoptosis in primary cultures of granule cells from mouse cerebellum.

The induction of neuronal cell death by DA *in vitro* is mediated by an increase in the production of reactive oxygen species (ROS) [6-8]. ROS are known to stimulate a number of events and pathways that lead to apoptosis, including mitogen-activated protein kinase (MAPK) signal transduction pathways [9]. In neuronal cells, p38 MAPK is preferentially activated by environmental stress and inflammatory cytokines, and it has been shown to promote neuronal cell death *in vitro*[10]. A recent study demonstrated that DA-induced cell death in primary neuronal cultures involves activation of p38 MAPK, and antioxidants prevented p38 MAPK phosphorylation [11]. Collectively, these lines of evidence suggest that DA induces ROS generation followed by p38 MAPK phosphorylation *in vitro*. In some studies, the hippocampus has been identified as a target site with high sensitivity to DA-induced toxicity [2,12,13]. A recent study demonstrated that abnormal ROS levels in the hippocampus of DA-treated mice activate the stress-activated protein kinase/c-Jun amino-terminal kinase (SAPK/JNK) pathway *in vivo*[14], but no studies have investigated ROS-mediated p38 MAPK signal transduction pathways in the hippocampus after administration of DA.

The WD40 repeat present in some proteins is a small structural motif of approximately 40 amino acids, typically bracketed by glycine-histidine and tryptophan-aspartate (GH-WD) [15]. Repeated WD40 motifs form a domain called the WD domain that is involved in protein-protein interactions. Proteins with WD40 repeats have important roles in a variety of cellular functions such as cell growth, proliferation, apoptosis, and intracellular signal transduction [15,16]. WD repeat-containing protein 35 (WDR35) is a novel member of this protein family [17,18]. In a mouse mutation screen for developmental phenotypes, Mill et al. [18] identified a mutation in the WDR35 gene as a cause of midgestation lethality associated with abnormalities characteristic of defects in the Hedgehog signaling pathway. Recently, we cloned rat WDR35, also referred to as naofen [19]. Very recently, we have found that bupivacaine, a local anesthetic, increases intracellular ROS levels and activates p38 MAPK in mouse neuroblastoma Neuro2a cells, resulting in apoptosis via an increase in the expression of WDR35 (under revision).

In the present study, we identified and quantified DA in the brains of rats after intraperitoneal DA administration by using liquid chromatography/ion trap tandem mass spectrometry (LC-MS/MS) analysis. We also performed histological examinations of the hippocampus after intraperitoneal DA administration in rats and investigated the potential involvement of glutamate receptors, ROS, p38 MAPK, and WDR35 in DA-induced toxicity.

## Results

### Identification and quantification of DA in the rat brain by LC-MS/MS analysis

**Figure 1:**
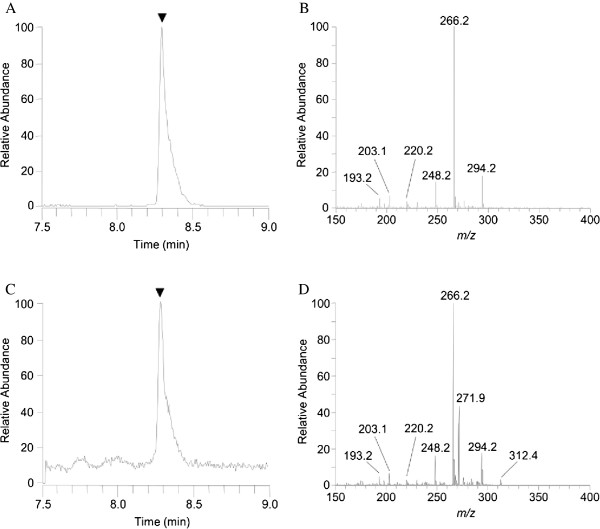
**LC-MS/MS analysis of domoic acid.** Intraperitoneally administered DA (1 mg/kg) was identified and quantified in the rat brain by LC-MS/MS analysis. **(A)** Accumulated reconstructed ion chromatograms of the three product ions (*m/z* 312 → 248, 266, 294) having a retention time of 8.3 min and **(B)** MS/MS spectrum for a 10 ng/ml standard solution of DA. **(C)** A brain sample from a rat taken at 30 min after intraperitoneal DA administration (1 mg/kg) showing the same retention time as that of the standard DA and **(D)** the presence of three diagnostic fragment ions.

LC-MS/MS analysis was performed to confirm that intraperitoneally administered DA reached the brain. The peak of the DA standard was detected at a retention time of 8.3 min under the LC conditions used in this experiment, and the full-scan mass spectrum showed the exclusive presence of the [M + H]^+^ ion at *m/z* 312 (data not shown). The MS/MS product ion spectra of DA, obtained using the [M + H]^+^ ion at *m/z* 312 as precursor, showed three fragment ions at *m/z* 248, *m/z* 266, and *m/z* 294. As an example, an accumulated reconstructed ion chromatogram (Figure 1A) of the three product ions (*m/z* 312 → 248, 266, 294) and the MS/MS spectrum (Figure 1B) for a 10 ng/ml standard DA solution are shown. Representative LC-MS/MS analysis of a brain sample from a rat obtained at 30 min after intraperitoneal injection of 1 mg/kg DA shows the same retention time (8.3 min) as that of the standard DA (Figure 1C) and the presence of three diagnostic fragment ions (Figure 1D) with the ion abundance ratios fully consistent with those of the standard DA, thus confirming the presence of DA in this brain sample.

Recovery tests were performed for quantification of the concentrations of DA in brain samples. The recovery of DA from brain samples spiked with 40 ng/g of DA was examined by calculating the ratio of the amount of analyte recovered after purification with the C18 cartridge column to the amount originally added. Overall recoveries and coefficients of variation were found to be satisfactory; these values were 84.4% and 11.0%, respectively (n = 3). The mean concentration of DA in rat brain samples obtained at 30 min after intraperitoneal administration of 1 mg/kg DA was 7.2 ± 0.75 ng/g (n = 3).

### Histological examination of the CA1 region of rat hippocampus following DA administration

**Figure 2:**
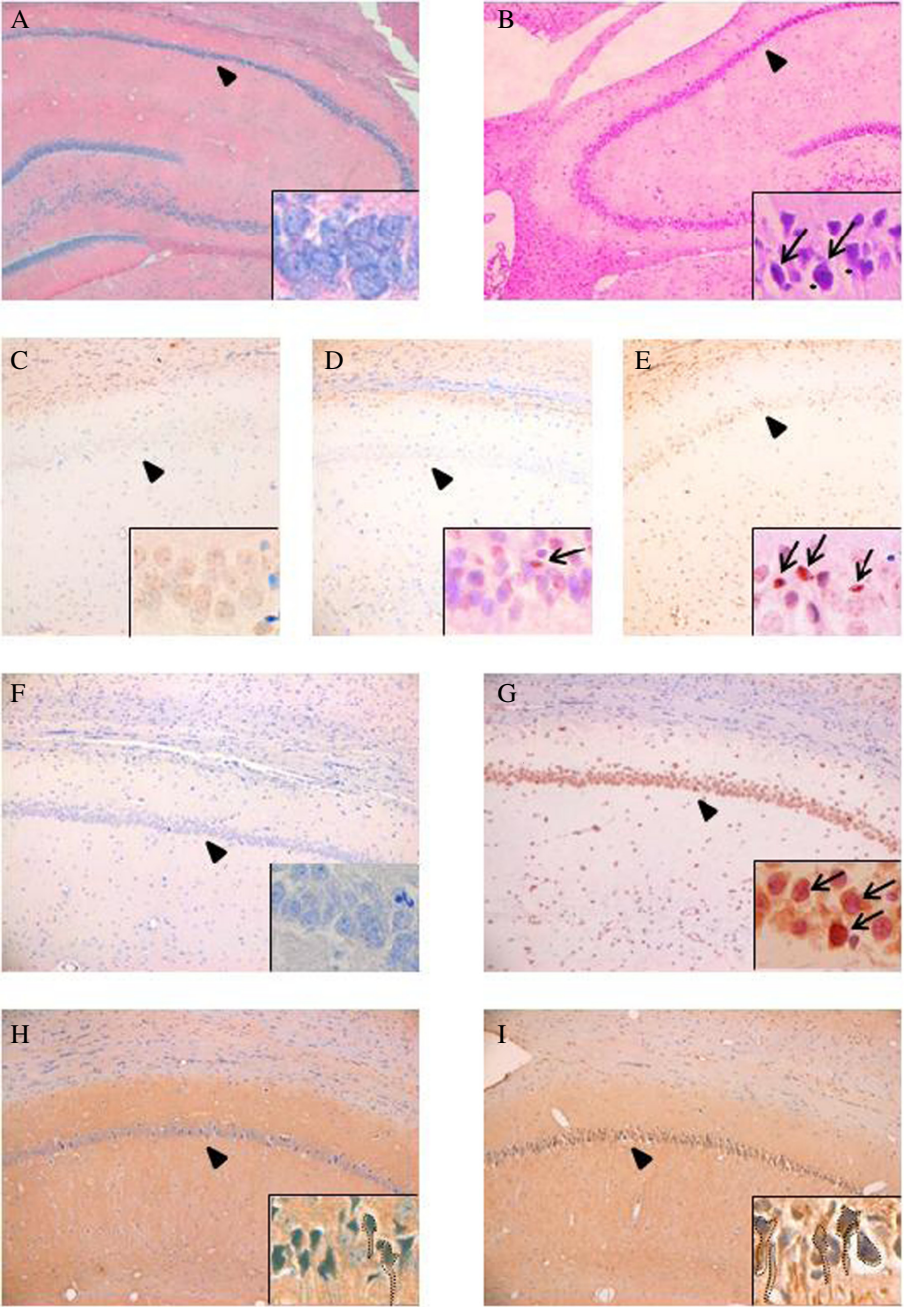
**Histological examination of the CA1 region of rat hippocampus following DA administration.** Rats were treated intraperitoneally with either the control (vehicle) or DA (1 mg/kg). Tissues were collected at 24 hours (A-D, F-I) and 5 days (E) after treatment and prepared for histology. Images are shown at × 50 (A, B) and × 100 (C-I) magnification. To clarify cell type-specific staining and subcellular staining, insets show the same samples (indicated by arrowheads) at × 400 magnification. (A, B) Hematoxylin-Eosin staining **(A)** 24 hours after vehicle administration and **(B)** 24 hours after DA administration. Arrow indicates neuronal shrinkage; asterisk indicates cell dropout. (C-E) TUNEL staining **(C)** 24 hours after vehicle administration, **(D)** 24 hours after DA administration, and **(E)** 5 days after DA administration. Arrow indicates TUNEL-positive neuron. (F, G) *In situ* hybridization **(F)** 24 hours after vehicle administration and **(G)** 24 hours after DA administration. Arrow indicates positive staining for WDR35 mRNA. (H, I) Immunohistochemical staining for WDR35 **(H)** 24 hours after vehicle administration and **(I)** 24 hours after DA administration. Positive staining neurons are enclosed by dotted lines.

Since LC-MS/MS analysis confirmed the distribution of intraperitoneally administered DA (1 mg/kg) to the brain, we carried out histological examinations of the CA1 region of the rat hippocampus at 24 hours after DA administration. Hematoxylin-Eosin staining showed no neurodegenerative changes such as neuronal shrinkage and cell dropout in the CA1 region after vehicle (saline) administration (Figure 2A). In contrast, neuronal shrinkage (arrow) and cell dropout (*) were observed after DA administration (Figure 2B). As Giordano et al. [7] reported that DA induces apoptosis in primary cultures of mouse neurons, we used TUNEL staining to examine whether intraperitoneal DA at 1 mg/kg induces apoptosis in the CA1 region of the rat hippocampus. TUNEL-positive neurons were not observed after vehicle administration (Figure 2C). In contrast, at 24 hours after DA administration, a few TUNEL-positive neurons were observed (Figure 2D). Furthermore, at 5 days after DA administration, many TUNEL-positive neurons were observed (Figure 2E). Administration of 1 mg/kg DA significantly increased the percentage of TUNEL positive cells at 24 hours (8.3 ± 1.3%, *P* < 0.05) and 5 days (19.0 ± 1.7%, *P* < 0.001) compared with that of vehicle (1.7 ± 1.1%). Expression of WDR35 mRNA in the CA1 region was not detected by *in situ* hybridization after administration of vehicle (Figure 2F), whereas it was observed in the soma of neurons after administration of DA (Figure 2G). Furthermore, immunohistochemical staining for WDR35 showed weak expression of WDR35 in the soma and axons of neurons (enclosed by the dotted lines) after administration of vehicle (Figure 2H) and strong expression of WDR35 in the soma and axons of neurons (enclosed by the dotted lines) after administration of DA (Figure 2I). These results indicated that DA induces the expression of WDR35 mRNA and protein in neurons in the CA1 region of the hippocampus.

### Quantitative examination of WDR35 expression in rat hippocampus following DA administration

**Figure 3:**
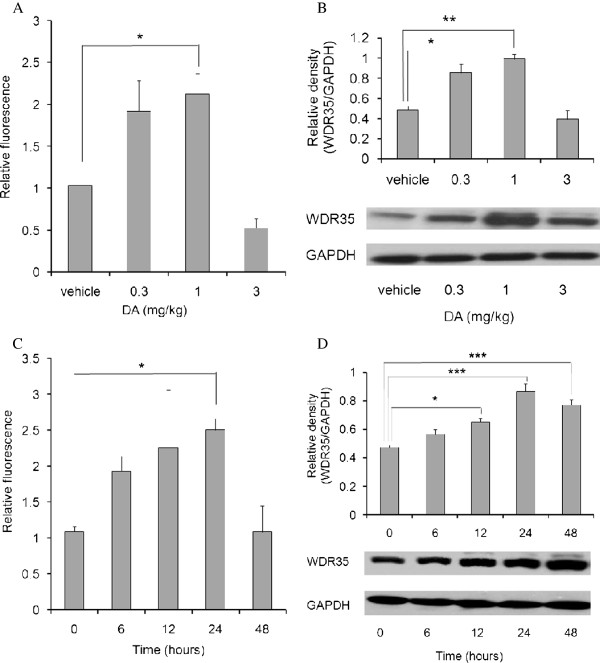
**Quantitative examination of WDR35 expression in rat hippocampus following DA administration.** (A, B) DA was administered to rats intraperitoneally at various doses. **(A)** WDR35 mRNA expression in hippocampal RNA preparations at 24 hours after DA administration was analyzed by qRT-PCR and expressed relative to the expression of GAPDH mRNA. **(B)** WDR35 protein expression in hippocampal homogenates at 24 hours after DA administration was analyzed by western blotting. (C, D) DA (1 mg/kg) was administered to rats intraperitoneally and WDR35 mRNA expression **(C)** and WDR35 protein expression **(D)** were determined in hippocampal samples collected at the times indicated after DA administration using the same methods as were used in (A) and (B). *P < 0.05, **P < 0.01 and ***P < 0.001 (n = 4).

Subsequently, real-time RT-PCR and western blotting analyses were performed to quantitatively examine the increase in WDR35 expression in the hippocampus following intraperitoneal DA administration. As shown in Figure 3A, administration of 1 mg/kg DA significantly increased WDR35 mRNA expression to a level 2.1-fold higher than the level observed after administration of vehicle. Furthermore, as shown in Figure 3B, administration of DA at 0.3 and 1 mg/kg significantly increased the expression of WDR35 protein compared with administration of vehicle. As the maximal effect was reached at 1 mg/kg DA, a dose of 1 mg/kg of DA was used for the following experiments.

As shown in Figure 3C, DA administration significantly increased the expression of WDR35 mRNA at 24 hours, when the level was 2.5-fold increased above the level at hour 0. Furthermore, as shown in Figure 3D, DA administration significantly increased the expression of WDR35 protein from 12 to 48 hours, with the maximal effect compared with hour 0 reached at 24 hours. These results showed that DA dose-dependently and time-dependently increases WDR35 expression in the rat hippocampus.

### Examination of the relationships between AMPA/KA receptors, ROS and WDR35

**Figure 4:**
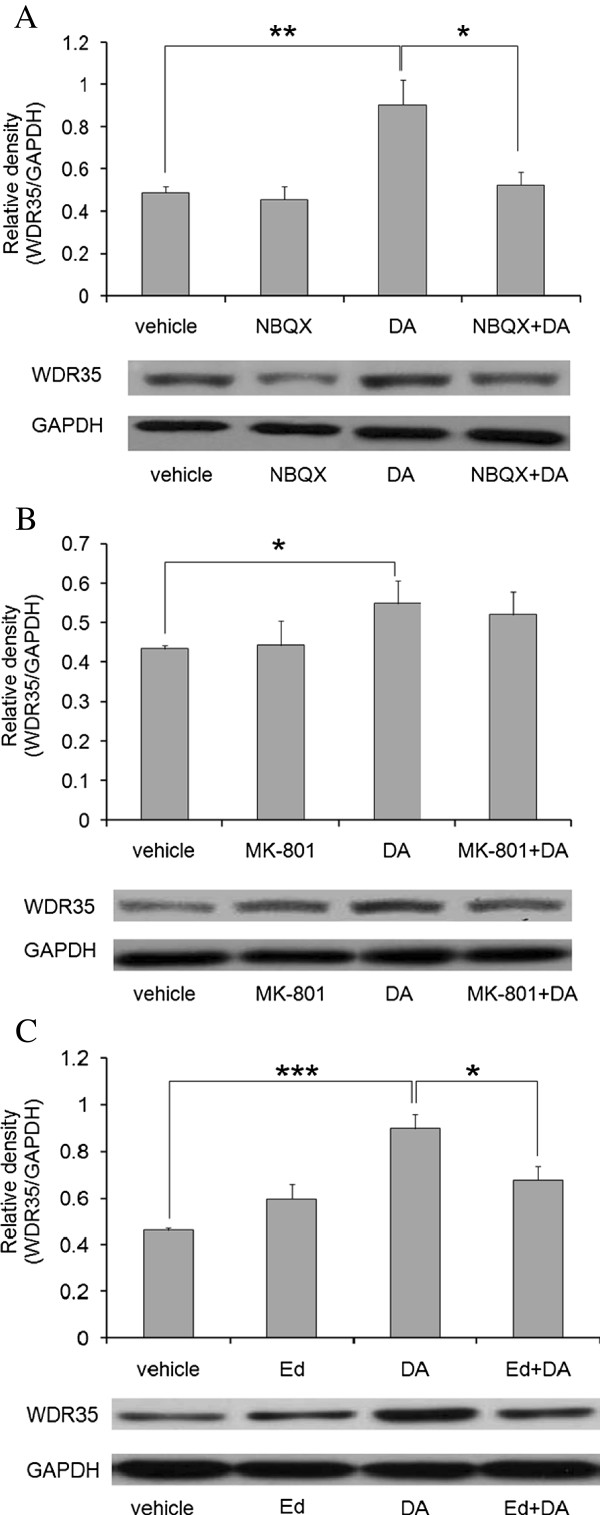
**Examination of the relationships between AMPA/KA receptors, ROS and WDR35.** Rats were administered glutamate receptor antagonists (3 mg/kg, i.p.) or edaravone (10 mg/kg, i.p.) at 1 hour before intraperitoneal administration of DA. WDR35 protein expression at 24 hours after DA administration in hippocampal samples from rats pretreated with **(A)** NBQX (AMPA/KA receptor antagonist), **(B)** MK-801 (NMDA receptor antagonist), or **(C)** edaravone (Ed, radical scavenger) was analyzed by western blotting. *P < 0.05, **P < 0.01 and ***P < 0.001 (n = 4).

Intraperitoneal injections of the selective glutamate receptor antagonists NBQX (AMPA/KA receptor antagonist, 3 mg/kg) and MK-801 (NMDA receptor antagonist, 3 mg/kg) were used to determine the types of glutamate receptors involved in the upregulation of WDR35 expression in the rat hippocampus at 24 hours after intraperitoneal DA administration (1 mg/kg). Analysis of immunoblots showed that NBQX significantly attenuated the DA-induced increase in WDR35 protein expression (Figure 4A). In contrast, MK-801 did not alter the DA-induced increase in WDR35 protein expression (Figure 4B). The antagonists alone had no effect on WDR35 protein expression.

Several reports have shown that DA induces ROS generation *in vitro* via activation of the AMPA/KA receptor [6-8]. Therefore, we examined whether ROS might play a role in the DA-induced increase in WDR35 protein expression in the rat hippocampus by using the radical scavenger edaravone (10 mg/kg, i.p.) that has been shown to exert neuroprotective effects in animal models of several brain disorders [20]. Analysis of immunoblots showed that pretreatment with edaravone significantly attenuated the DA-induced upregulation of WDR35 (Figure 4C).

### Examination of the relationships between AMPA/KA receptors, ROS and p38 MAPK

**Figure 5:**
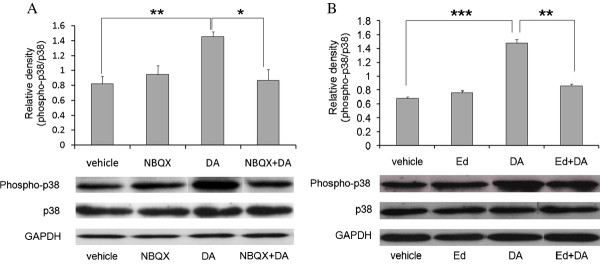
**Examination of the relationships between AMPA/KA receptors, ROS and p38 MAPK.** Rats were administered **(A)** NBQX (3 mg/kg, i.p.) or **(B)** edaravone (10 mg/kg, i.p.) at 1 hour before intraperitoneal administration of DA. Expression of p38 and phospho-p38 MAPK in rat hippocampus at 24 hours after intraperitoneal DA administration was analyzed by western blotting. *P < 0.05, **P < 0.01 and ***P < 0.001 (n = 3-7).

As Harper et al. [10] reported that ROS activates the p38 MAPK pathway, we then investigated whether intraperitoneal DA administration increases p38 MAPK phosphorylation in the rat hippocampus. Treatment with DA significantly increased p38 MAPK phosphorylation in immunoblots of hippocampal tissue samples (Figure 5A, 5B). We also examined the effect of NBQX and edaravone on the DA-induced increase in p38 MAPK phosphorylation in the rat hippocampus. NBQX and edaravone significantly attenuated DA-induced p38 MAPK phosphorylation in immunoblots of hippocampal tissue samples (Figure 5A, 5B). No significant change in the expression of p38 MAPK protein was observed. These results suggested that activation of the AMPA/KA receptor resulting in ROS generation followed by p38 MAPK phosphorylation might be involved in the DA-induced upregulation of WDR35.

## Discussion

LC-MS/MS has emerged as a powerful technique for the identification and quantification of natural products, such as toxins, in complicated matrices. In the present study, we applied this technique to the analysis of DA in the rat brain following an intraperitoneal dose of 1 mg/kg DA and confirmed the presence of DA (Figure 1) at a mean concentration of 7.2 ng/g tissue. A recent study using LC-MS/MS reported that the concentration of DA in fetal rat brains following an intravenous dose of 1 mg/kg DA in pregnant rats was stable at a mean of 8.12 ng/g tissue [21]. Although the experimental conditions differed greatly between this study and ours, the concentration of DA achieved in the brains of rats appeared to be comparable.

In the present study, neuronal shrinkage and cell dropout were observed in the CA1 region of the rat hippocampus at 24 hours after DA administration (Figure 2B). Similar neurodegenerative changes were also observed in rat brain in previous reports, although the doses of DA, route of administration of DA, and time course of measured effects were different in these studies: 2 mg/kg i.p. at 6–24 hours [22], 1.32 mg/kg i.p. at 8 days [23], and 0.75 mg/kg i.v. at 5–21 days [24]. As reported previously [8,24], TUNEL-positive cells were observed following DA administration (Figure 2D, 2E). These observations suggest that in the present study, intraperitoneally administered DA induced neurodegenerative changes including apoptosis in the hippocampus.

Previously, we reported that enhanced WDR35 expression may mediate apoptosis in several animal models [25,26]. As expected, expression of WDR35 was observed in neurons in the CA1 region of the rat hippocampus following DA administration (Figure 2G, 2I). Quantitative examination of WDR35 expression showed that WDR35 is upregulated in the rat hippocampus following DA administration. The increases in WDR35 mRNA and protein expression in the hippocampus were both dose-dependent (Figure 3A, 3B) and time-dependent (Figure 3C, 3D), suggesting that the increased expression of WDR35 may participate in the DA-induced neurodegenerative changes that were observed.

In studies *in vitro*, the toxicity of DA is mediated mainly via activation of the AMPA/KA receptor [3-5], whereas neurotoxicity is mediated secondarily via DA activation of the NMDA receptor/Ca^2+^ pathway [27,28]. We examined the role of glutamate receptors in the DA-induced increase in WDR35 expression by using pharmacological blocking agents. We demonstrated that an AMPA/KA receptor antagonist (NBQX) significantly attenuated the DA-induced increase in WDR35 protein expression *in vivo* (Figure 4A), but an NMDA receptor antagonist (MK-801) did not (Figure 4B). These results suggest that the AMPA/KA receptor could play a role in DA-induced increases in WDR35 expression *in vivo*.

The induction of neuronal cell death by DA is mediated by increased ROS production [6-8]. In addition, kainic acid has been reported to stimulate ROS generation [6]. These observations led us to investigate the relationship between ROS and WDR35 expression by using a radical scavenger, edaravone. We demonstrated that edaravone significantly attenuated DA-induced WDR35 expression (Figure 4C), suggesting that DA-induced ROS production may increase the expression of WDR35 *in vivo*. In some studies, ROS have been shown to activate p38 MAPK, eventually causing apoptosis [10]. In the present study, we demonstrated that DA significantly increased p38 MAPK phosphorylation *in vivo*, and this effect was significantly attenuated by NBQX and edaravone (Figure 5A, 5B). Very recently, we found that the local anesthetic bupivacaine increases intracellular ROS levels and activates p38 MAPK, resulting in apoptosis via an increase in WDR35 expression in mouse neuroblastoma Neuro2a cells (under revision). Taking these observations into account, we speculate that in the present study, the increase in ROS production and activation of p38 MAPK by DA resulted in increased expression of WDR35 in the rat hippocampus.

Although several studies demonstrated that DA induces excitotoxic neuronal damage via activation of glutamate receptors [8,24], the mechanistic cascades have not been investigated *in vivo*. In the present study, we provided the first evidence of the sequence of mechanisms for DA-induced toxicity by histological and biochemical examinations *in vivo*, in which DA activated the AMPA/KA receptor, and induced ROS production and p38 MAPK phosphorylation, resulting in an increase in the expression of WDR35. Our findings will be useful for future studies such as possible involvement of transcription factors of activating protein-1 (AP-1) and nuclear factor-kappa B (NF-κB) families in DA-induced toxicity, and detailed sequence of events including the role of WDR35.

## Conclusions

In conclusion, our results indicated that DA activated the AMPA/KA receptor, and induced ROS production and p38 MAPK phosphorylation, resulting in an increase in the expression of WDR35.

## Methods

### Reagents

Domoic acid (Sigma, St. Louis, MO, USA) was dissolved in saline solution (0.9% NaCl) at 1 mg/ml. The glutamate receptor antagonists, 2,3-dioxo-6-nitro-7-sulfamoylbenzo[f]quinoxaline (NBQX, Sigma, St. Louis, MO, USA) and dizocilpine maleate (MK-801, Tocris Bioscience, Ellisville, MO, USA) were dissolved in saline solution at 3 mg/ml. Edaravone (Tocris Bioscience, Ellisville, MO, USA) was dissolved in dimethyl sulfoxide at 10 mg/ml.

### Animals and treatments

Eight-week-old male Sprague–Dawley rats weighing approximately 300 g were purchased from SLC (Shizuoka, Japan) and habituated to the laboratory for 1 week before the experiments began. All procedures were approved by the Animal Care Committee of Aichi Medical University. All rats were kept in a climate-controlled room under 12/12-hour light–dark cycles with free access to food and tap water throughout the studies.

For the mass spectrometric analysis, rats were administered DA intraperitoneally at a dose of 1 mg/kg. Thirty minutes later, the rats were deeply anesthetized with pentobarbital sodium and were then perfused transcardially with cold phosphate-buffered saline (PBS). The brains were then removed and weighed. For histological examinations, rats were administered DA (1 mg/kg) or vehicle intraperitoneally. At 24 hours or 5 days after DA or vehicle administration, rats were deeply anesthetized with pentobarbital sodium and perfused transcardially with cold PBS followed by cold 4% paraformaldehyde in PBS, pH 7.2. The brains were removed and postfixed with 4% paraformaldehyde at 4°C for 24 h, then were dehydrated and embedded in paraffin. The paraffin blocks were stored at 4°C. Sections were cut at a thickness of 4 μm and processed for Hematoxylin-Eosin staining, *in situ* hybridization, immunohistochemistry, and TUNEL staining. For the qRT-PCR and western blot experiments, DA or vehicle administered rats were sacrificed by decapitation. The brains were rapidly excised, and hippocampi were rapidly dissected on an ice-cold dissection board. For dose–response studies, rats were administered DA intraperitoneally at doses of 0.3, 1, and 3 mg/kg and were sacrificed 24 hours later. For time-course studies, rats were sacrificed at 0, 6, 12, 24, and 48 hours after intraperitoneal administration of 1 mg/kg DA. Seizures [29] were observed in 3 mg/kg group. For pretreatment studies, NBQX, MK-801, and edaravone were administered to rats intraperitoneally at one hour before DA administration, and the rats were sacrificed at 24 hours after DA administration. Control rats received an equivalent volume of vehicle.

### Preparation of extracts from rat brains for mass spectrometric analysis

Isolated rat brains were minced in 50% aqueous methanol (8 ml), homogenized, and centrifuged at 10,000 *g* for 20 min. The supernatant was passed through a cartridge column of Sep-Pak^®^ Plus C18 (Waters Co., Milford, MA, USA), and filtered with Amicon^®^ Ultra 0.5 ml 10 K centrifugal filter devices (Millipore Co., Billerica, MA, USA). The filtrate was subjected to LC-MS/MS analysis.

### LC-MS/MS

The LC separation was performed using an ACCELA HPLC system (Thermo Fisher Scientific, Waltham, MA, USA). Separation was accomplished with a TSK-GEL ODS-80Ts column (150 × 2.0 mm, Tosoh, Tokyo, Japan) at 30°C. Water containing 0.1% formic acid was used as eluent A, and acetonitrile containing 0.1% formic acid was used as eluent B. The LC elution gradient employed was: 0 to 5 min 100% A, 5 to 10 min eluent A decreased linearly to 50%, and 10 to 12 min 100% B. The eluent shifted back to 100% A and was held for 3 min to equilibrate the column for the next sample. The flow rate was 0.5 ml/min. The MS analysis was accomplished using a LTQ Velos spectrometer (Thermo Fisher Scientific, San Jose, CA, USA) equipped with a heated electrospray ionization (HESI) source. Positive ion HESI was chosen for the detection of DA. The source voltage was set at 3 kV, the capillary temperature was set at 350°C, and the tube lens offset was set at 15 V. The sheath gas flow rate was 35 (arbitrary units) and the auxiliary gas flow rate was 10 (arbitrary units). Full scan experiments were performed in the range *m/z* 50–1000. The total number of microscans was set at 1 and the maximum injection time at 10 ms. Subsequent MS/MS experiments were performed in the range *m/z* 85–400. The maximum injection time was set at 100 ms. Reproducible calibration curves for DA were obtained with correlation coefficients greater than 0.999 (known concentration versus analyte), and the curves were linear over the range of 0.2-10 ng/ml.

### *In situ* hybridization

The sequence of the WDR35 probe was 5^′^AAGCACAAACTGAGGGTGATTTTCATCAGC-3^′^. Hybridization was performed at 105°C for 5 minutes and 50°C for 3 hours, with the probe diluted at 1:1000. The hybridization signal was amplified with the TSA Biotin System (PerkinElmer, Waltham, MA, USA) and visualized with DAB (Falma, Tokyo, Japan). The sections were counterstained with hematoxylin as described previously [25].

### Immunohistochemical staining for WDR35

Rat brain sections were treated with 3% H_2_O_2_ in methanol for 30 min to inactive endogenous peroxidase, blocked at room temperature for 60 min with 4% goat serum albumin in PBS, and rinsed in 0.01 M PBS with 0.1% Tween20^®^ (PBS-T). Sections were then incubated at room temperature overnight with anti-WDR35 antibody (1:1000, Abcam, Cambridge, UK) in PBS-T. Immunohistochemical staining was done according to the manufacturer’s protocol using a Vectastain ABC *Elite* kit (Vector Laboratories Inc., Burlingame, CA, USA) with the 3,3^′^-diaminobenzidine (DAB) reaction. Sections were counterstained with hematoxylin. Sections with no primary antibody were included as negative controls to verify the secondary antibody specificity.

### Terminal deoxynucleotidyl transferase-mediated dUTP nick end-labeling (TUNEL) assay

TUNEL was performed using the ApopTag^®^ Plus In Situ Apoptosis Detection Kit (Chemicon International, Temecula, CA, USA) according to the manufacturer’s instructions and visualized with the TSA Biotin System and DAB. The sections were counterstained with 0.5% methyl green (Wako Pure Chemicals, Osaka, Japan). Apoptotic and nonapoptotic cells were counted in three randomly chosen microscopic fields, and results are expressed as percentage of apoptotic cells ± S.E.

### Quantitative real-time reverse transcriptase-polymerase chain reaction (qRT-PCR)

Total RNA (1 μg) was extracted from rat hippocampus homogenate with TRIzol^®^ reagent (Invitrogen, Carlsbad, CA, USA) and reverse transcribed with a ReverTra Ace qPCR RT kit (Toyobo, Osaka, Japan). Quantitative RT-PCR was performed as described previously [26] with the ABI StepOne Plus real-time PCR system and a Taqman Gene Expression Assay (Applied Biosystems, Tokyo, Japan) according to the manufacturer’s instructions. WDR35 mRNA levels were quantified relative to GAPDH mRNA levels, the internal control. The results are presented according to the ΔΔC_T_ method as ratios of the target to the internal control as described previously [26].

### Western blot analysis

Rat hippocampi were homogenized in lysis buffer [62.5 mM Tris–HCl (pH6.8), 2.5% SDS, 10% glycerol] containing a protease inhibitor cocktail (Roche Applied Sciences, Mannheim, Germany) and heated in boiling water for 5 min. After centrifugation, the supernatants were collected, and the protein concentration of samples was determined by the DC protein assay (Bio-Rad Laboratories, Hercules, CA, USA). Samples (30 μg protein) were mixed with 2-mercaptoethanol and Bromo Phenol Blue buffer, separated by SDS-PAGE, and transferred to PVDF membranes (Millipore Corporation, Billerica, MA, USA). The blots were incubated with rabbit antibodies against WDR35 (132kDa, 1:1000; Abcam, Cambridge, UK), p38 MAPK (43 kDa, 1:1000; Cell Signaling Technology, Danvers, MA, USA), phospho-p38 MAPK (43 kDa, 1:1000; Cell Signaling Technology) or GAPDH (37 kDa, 1:20,000; Cell Signaling Technology) followed by peroxidase-conjugated anti-rabbit IgG (1:5000; Zymed Laboratories, San Francisco, CA, USA). The apparent molecular weight of each protein was determined by BlueStar Prestained Protein Marker (Nippon Genetics Europe GmbH, Dueren, Germany). Detection was performed with the ECL Prime Western Blotting Reagent (GE Healthcare, Buckinghamshire, UK). Protein levels were quantified by densitometric scanning and expressed as the ratio to GAPDH, as described previously [30].

### Statistical analysis

All results were expressed as mean ± standard error of the mean (SEM). Data were analyzed for statistical significance with one-way analysis of variance (ANOVA), followed by post hoc analysis with the Bonferroni method. Differences were considered significant at *P* < 0.05.

## Competing interests

The authors declare that they have no competing interests.

## Authors’ contributions

KT carried out the histological and biochemical studies, performed the statistical analysis, and drafted the manuscript. FK carried out the LC-MS/MS study, performed the statistical analysis, participated in the study design, drafted and critically appraised the manuscript. TO participated in the histological studies and helped to draft the manuscript. GGF and LH participated in the biochemical experiments and contributed to data acquisition and analysis. NI was involved in the conceptualization of the study. SO conceived of the study, and participated in its design and coordination and helped to draft the manuscript. All authors read and approved the final manuscript.

## References

[B1] Sari P Kerr DS Domoic acid-induced hippocampal CA1 hyperexcitability independent of region CA3 activity Epilepsy Res 2001 47 1–2 65 76 11673022 10.1016/s0920-1211(01)00295-9

[B2] Perez-Gomez A Tasker RA Enhanced neurogenesis in organotypic cultures of rat hippocampus after transient subfield-selective excitotoxic insult induced by domoic acid Neuroscience 2012 208 97 108 22366222 10.1016/j.neuroscience.2012.02.003

[B3] Novelli A Kispert J Fernandez-Sanchez MT Torreblanca A Zitko V Domoic acid-containing toxic mussels produce neurotoxicity in neuronal cultures through a synergism between excitatory amino acids Brain Res 1992 577 1 41 48 10.1016/0006-8993(92)90535-H 1355695

[B4] Hogberg HT Bal-Price AK Domoic acid-induced neurotoxicity is mainly mediated by the AMPA/KA receptor: comparison between immature and mature primary cultures of neurons and glial cells from rat cerebellum J Toxicol 2011 2011 543512 22135676 10.1155/2011/543512 PMC3216357

[B5] Giordano G White CC Mohar I Kavanagh TJ Costa LG Glutathione levels modulate domoic acid induced apoptosis in mouse cerebellar granule cells Toxicol Sci 2007 100 2 433 444 10.1093/toxsci/kfm236 17804861

[B6] Bondy SC Lee DK Oxidative stress induced by glutamate receptor agonists Brain Res 1993 610 2 229 233 10.1016/0006-8993(93)91405-H 8319085

[B7] Giordano G White CC McConnachie LA Fernandez C Kavanagh TJ Costa LG Neurotoxicity of domoic acid in cerebellar granule neurons in a genetic model of glutathione deficiency Mol Pharmacol 2006 70 6 2116 2126 10.1124/mol.106.027748 17000861

[B8] Xu R Tao Y Wu C Yi J Yang Y Yang R Hong D Domoic acid induced spinal cord lesions in adult mice: evidence for the possible molecular pathways of excitatory amino acids in spinal cord lesions Neurotoxicology 2008 29 4 700 707 10.1016/j.neuro.2008.04.011 18534681

[B9] Cuadrado A Nebreda AR Mechanisms and functions of p38 MAPK signalling Biochem J 2010 429 3 403 417 10.1042/BJ20100323 20626350

[B10] Harper SJ LoGrasso P Signalling for survival and death in neurones: the role of stress-activated kinases, JNK and p38 Cell Signal 2001 13 5 299 310 10.1016/S0898-6568(01)00148-6 11369511

[B11] Giordano G Klintworth HM Kavanagh TJ Costa LG Apoptosis induced by domoic acid in mouse cerebellar granule neurons involves activation of p38 and JNK MAP kinases Neurochem Int 2008 52 6 1100 1105 10.1016/j.neuint.2007.11.004 18164102 PMC2394507

[B12] Strain SM Tasker RA Hippocampal damage produced by systemic injections of domoic acid in mice Neuroscience 1991 44 2 343 352 10.1016/0306-4522(91)90059-W 1944890

[B13] Gill DA Ramsay SL Tasker RA Selective reductions in subpopulations of GABAergic neurons in a developmental rat model of epilepsy Brain Res 2010 1331 114 123 20331981 10.1016/j.brainres.2010.03.054

[B14] Wu DM Lu J Zheng YL Zhang YQ Hu B Cheng W Zhang ZF Li MQ Small interfering RNA-mediated knockdown of protein kinase C zeta attenuates domoic acid-induced cognitive deficits in mice Toxicol Sci 2012 128 1 209 222 10.1093/toxsci/kfs124 22474074

[B15] Neer EJ Schmidt CJ Nambudripad R Smith TF The ancient regulatory-protein family of WD-repeat proteins Nature 1994 371 6495 297 300 10.1038/371297a0 8090199

[B16] Smith TF Gaitatzes C Saxena K Neer EJ The WD repeat: a common architecture for diverse functions Trends Biochem Sci 1999 24 5 181 185 10.1016/S0968-0004(99)01384-5 10322433

[B17] Gilissen C Arts HH Hoischen A Spruijt L Mans DA Arts P van Lier B Steehouwer M van Reeuwijk J Kant SG Roepman R Knoers NV Veltman JA Brunner HG Exome sequencing identifies WDR35 variants involved in Sensenbrenner syndrome Am J Hum Genet 2010 87 3 418 423 10.1016/j.ajhg.2010.08.004 20817137 PMC2933349

[B18] Mill P Lockhart PJ Fitzpatrick E Mountford HS Hall EA Reijns MA Keighren M Bahlo M Bromhead CJ Budd P Aftimos S Delatycki MB Savarirayan R Jackson IJ Amor DJ Human and mouse mutations in WDR35 cause short-rib polydactyly syndromes due to abnormal ciliogenesis Am J Hum Genet 2011 88 4 508 515 10.1016/j.ajhg.2011.03.015 21473986 PMC3071922

[B19] Feng GG Li C Huang L Tsunekawa K Sato Y Fujiwara Y Komatsu T Honda T Fan JH Goto H Koide T Hasegawa T Ishikawa N Naofen, a novel WD40-repeat protein, mediates spontaneous and tumor necrosis factor-induced apoptosis Biochem Biophys Res Commun 2010 394 1 153 157 10.1016/j.bbrc.2010.02.133 20193664

[B20] Ohta M Higashi Y Yawata T Kitahara M Nobumoto A Ishida R Tsuda M Fujimoto Y Shimizu K Attenuation of axonal injury and oxidative stress by edaravone protects against cognitive impairments after traumatic brain injury Brain Res 2012 Epub ahead of print 10.1016/j.brainres.2012.09.011 22982593

[B21] Fuquay JM Muha N Wang Z Ramsdell JS Toxicokinetics of domoic acid in the fetal rat Toxicology 2012 294 1 36 41 10.1016/j.tox.2012.01.012 22306965

[B22] Tryphonas L Truelove J Nera E Iverson F Acute neurotoxicity of domoic acid in the rat Toxicol Pathol 1990 18 1 Pt 1 1 9 2362984 10.1177/019262339001800101

[B23] Sobotka TJ Brown R Quander DY Jackson R Smith M Long SA Barton CN Rountree RL Hall S Eilers P Johannessen JN Scallet AC Domoic acid: neurobehavioral and neurohistological effects of low-dose exposure in adult rats Neurotoxicol Teratol 1996 18 6 659 670 10.1016/S0892-0362(96)00120-1 8947943

[B24] Ananth C Thameem Dheen S Gopalakrishnakone P Kaur C Domoic acid-induced neuronal damage in the rat hippocampus: changes in apoptosis related genes (bcl-2, bax, caspase-3) and microglial response J Neurosci Res 2001 66 2 177 190 10.1002/jnr.1210 11592113

[B25] Sato Y Feng GG Huang L Fan JH Li C An J Tsunekawa K Kurokawa S Fujiwara Y Komatsu T Kondo F Ishikawa N Enhanced expression of naofen in kidney of streptozotocin-induced diabetic rats: possible correlation to apoptosis of tubular epithelial cells Clin Exp Nephrol 2010 14 3 205 212 10.1007/s10157-010-0276-1 20224876

[B26] Fan JH Feng GG Huang L Tsunekawa K Honda T Katano Y Hirooka Y Goto H Kandatsu N Ando K Fujiwara Y Koide T Okada S Ishikawa N Role of naofen in apoptosis of hepatocytes induced by lipopolysaccharide through mitochondrial signaling in rats Hepatol Res 2012 42 7 696 705 10.1111/j.1872-034X.2012.00972.x 22409254

[B27] Berman FW Murray TF Domoic acid neurotoxicity in cultured cerebellar granule neurons is mediated predominantly by NMDA receptors that are activated as a consequence of excitatory amino acid release J Neurochem 1997 69 2 693 703 9231729 10.1046/j.1471-4159.1997.69020693.x

[B28] Berman FW LePage KT Murray TF Domoic acid neurotoxicity in cultured cerebellar granule neurons is controlled preferentially by the NMDA receptor Ca(2+) influx pathway Brain Res 2002 924 1 20 29 10.1016/S0006-8993(01)03221-8 11743991

[B29] Fuquay JM Muha N Pennington PL Ramsdell JS Domoic acid induced status epilepticus promotes aggressive behavior in rats Physiol Behav 2012 105 2 315 320 10.1016/j.physbeh.2011.08.013 21875611

[B30] An J Feng GG Huang L Kurokawa T Nonami T Koide T Kondo F Komatsu T Tsunekawa K Fujiwara Y Goto H Nishikawa H Miki T Sugiyama S Ishikawa N Effects of 1-O-hexyl-2,3,5-trimethylhydroquinone on carbon tetrachloride-induced hepatic cirrhosis in rats Hepatol Res 2010 40 6 613 621 10.1111/j.1872-034X.2010.00638.x 20412328

